# Inflammatory Bowel Disease in Kazakhstan: Phenotypes, Disease Activity, and Treatment Patterns from a National Registry

**DOI:** 10.3390/jcm15176851

**Published:** 2026-09-04

**Authors:** Jamilya Kaibullayeva, Aliya Ualiyeva, Ainash Tanabayeva, Assyltay Nauryzbayeva, Nadezhda Kabdulina, Zukhra Agzamova, Yelena Gladysheva, Ayagoz Yeskozhina, Issa Mussatayev, Ainur Doszhan, Assem Kurmangaliyeva, Elvira Kenzhebayeva, Dariga Kushimova, Yelena Laryushina, Zhasulan Temirbayev, Lyudmila Rayushkina, Mira Izenova, Zamzagul Smagulova, Ainur Chalabayeva, Raushan Dossanova, Dana Adayeva, Polina Muldasheva, Roza Atasheva, Yelena Kravtsova, Salavat Kuldeyev, Oksana Shchukina

**Affiliations:** 1 Department of Gastroenterology, Research Institute of Cardiology and Internal Diseases, Almaty 050000, Kazakhstan; kaibullaev@mail.ru (J.K.);; 2 Department of Epidemiology, Biostatistics, and Evidence-Based Medicine, Farabi University, Almaty 050040, Kazakhstan; 3 Department of Therapy, National Scientific Medical Center, Astana 010009, Kazakhstan; 4 Department of Therapy, Regional Clinical Hospital, Karaganda 100000, Kazakhstan; 5 Department of Gastroenterology, LLP Medico–Diagnostic Center, Pavlodar 140000, Kazakhstan; 6 Department of Therapy, Multidisciplinary Regional Hospital, Petropavlovsk 150010, Kazakhstan; 7 Department of Gastroenterology, Medical Center GASTROMED, Shymkent 160005, Kazakhstan; 8 Department of Gastroenterology, Aktobe Medical Center, Aktobe 030000, Kazakhstan; 9 Department of Gastroenterology, West Kazakhstan Medical University Named After M. Ospanov, Aktobe 030019, Kazakhstan; 10 Department of Gastroenterology, Karaganda Medical University, Karaganda 100000, Kazakhstan; 11 Therapy Department, City Polyclinic № 6, Taraz 080016, Kazakhstan; 12 Therapy Department, Multidisciplinary Regional Hospital, Kyzylorda 120008, Kazakhstan; 13 Therapy Department, Pavlodar Regional Hospital Named After G. Sultanov, Pavlodar 140010, Kazakhstan; 14 Therapy Department, City Diagnostic Center, Shymkent 160021, Kazakhstan; 15 Therapy Department, Akmola Regional Hospital, Kokshetau 020000, Kazakhstan; 16 Therapy Department, Regional Hospital, Shymkent 160011, Kazakhstan; 17 Therapy Department, Mangystau Regional Multidisciplinary Hospital, Aktau 130000, Kazakhstan; 18 Therapy Department, Outpatient Center, Ust-Kamenogorsk 070002, Kazakhstan; 19 Therapy Department, Kostanay Regional Hospital, Kostanay 110000, Kazakhstan; 20 Inflammatory Bowel Disease Unit, Academician I.P. Pavlov First Saint Petersburg State Medical University, Saint Petersburg 197022, Russia

**Keywords:** inflammatory bowel disease, ulcerative colitis, Crohn’s disease, treat-to-target, clinical outcomes, registry

## Abstract

**Background/Objectives:** Inflammatory bowel diseases (IBDs) are an increasingly important global health problem, yet Central Asia remains underrepresented in international IBD data. To evaluate the clinical and demographic characteristics of patients included in the national multicenter clinical IBD registry of the Republic of Kazakhstan (IBD Registry) according to disease activity at registry enrollment visit and to identify factors associated with active IBD at that time. **Methods**: We conducted a cross-sectional analysis of the registry enrollment visit data from the national multicenter clinical IBD registry of the Republic of Kazakhstan. The study included patients with ulcerative colitis (UC) and Crohn’s disease (CD) registered between 2021 and 2024. Disease activity at the registry enrollment visit was assessed using the full Mayo score for UC and the Harvey–Bradshaw index for CD. Univariable and multivariable logistic regression analyses were performed to identify factors associated with active disease. **Results:** The analysis included 1521 patients with IBD, 68.4% with ulcerative colitis and 31.6% with Crohn’s disease. At the registry enrollment visit, 77.5% of patients had clinically active disease. In UC, active disease was independently associated with rural residence (aOR 2.11; 95% CI 1.25–3.56), ≥2 exacerbations per year (aOR 3.47; 95% CI 2.18–5.51), current corticosteroid therapy (aOR 5.96; 95% CI 3.04–11.69), and immunosuppressive therapy (aOR 2.71; 95% CI 1.15–6.38), whereas rare exacerbations were associated with lower odds of activity (aOR 0.41; 95% CI 0.23–0.73). In CD, active disease was independently associated with ≥2 exacerbations per year (aOR 2.41; 95% CI 1.47–3.95), colonic (L2) location (aOR 2.32; 95% CI 1.25–4.30), ileocolonic (L3) location (aOR 2.24; 95% CI 1.25–4.04), extraintestinal manifestations (aOR 2.88; 95% CI 1.68–4.93), and current corticosteroid therapy (aOR 1.77; 95% CI 1.03–3.05), whereas immunosuppressive therapy was associated with lower odds of active disease (aOR 0.46; 95% CI 0.24–0.89). Biologic therapy was not independently associated with disease activity in either UC or CD. **Conclusions**: A high proportion of patients treated at participating secondary and tertiary centers had clinically active IBD at registry enrollment. The profiles of factors associated with activity differed between UC and CD, while frequent exacerbations were consistently associated with active disease in both conditions. These findings should be interpreted as cross-sectional associations rather than causal or prognostic relationships, particularly for treatment-related factors.

## 1. Introduction

Inflammatory bowel diseases (IBDs) impose a substantial burden on healthcare systems around the world. Over the past 10 years, the understanding of the epidemiology of inflammatory bowel diseases has changed substantially. While ulcerative colitis and Crohn’s disease were once considered illnesses mostly common in Western countries, today their prevalence is rapidly increasing and becoming a global problem in countries in Asia, Africa, and Latin America [1].

The four-stage epidemiological model proposed by G. Kaplan and the GIVES-21 consortium describes the evolution of IBD from rare cases to a significant chronic burden on the healthcare system [2]. Currently in Asia, there is a steady increase in prevalence and incidence, linked to rapid urbanization and changes in habits and lifestyle [1,3]. As for Kazakhstan, the country is currently in the acceleration stage, characterized by a sharp rise in incidence and following the epidemiological path of Western countries in the mid-20th century [2]. As more IBD patients accumulate, an increase in disease prevalence and pressure on the healthcare system is expected [2]. In this context, national and international IBD registries (UK IBD Registry, SWIBREG, ENEIDA, etc.) play a pivotal role, providing continuous collection of standardized clinical data from routine practice. Their development has become the basis for expanding our understanding of the phenotypic diversity of IBD, the natural course of the disease, and the effectiveness and safety of various treatment strategies [4,5,6].

In Kazakhstan, information on the prevalence of IBD is traditionally based on official statistics of the Ministry of Health, which shows that the rate for ulcerative colitis is 31.5, and for Crohn’s disease—6.3 cases per 100,000 people [7]. However, population-based study published in 2020 by Kazakhstani researchers have demonstrated that the actual number of patients could be several times higher than officially reported: the overall prevalence of the disease was 113.9 per 100,000 people, ulcerative colitis–84.4 per 100,000, and Crohn’s disease–29.5 per 100,000 people [7]. Despite the fact that epidemiological studies [7,8] and research on the organization of IBD care [9,10] conducted in our country had significantly expanded our understanding of the epidemiology and burden of IBD in Kazakhstan, information on clinical profile of the disease, complications, surgical interventions and routine clinical management is still limited.

In addition to these epidemiological gaps, the national clinical context of IBD management should also be considered. In Kazakhstan, patients with IBD are managed according to national clinical protocols for ulcerative colitis [11] and Crohn’s disease [12]. Treatment options include 5-aminosalicylic acid drugs, corticosteroids, immunosuppressive, and biological therapy; the choice of treatment is based on the activity and phenotype of the disease, the presence of extraintestinal manifestations, comorbidities and other individual clinical characteristics of the patient, as well as the response to previous therapy. Importantly, 5-aminosalicylates and biologic therapies are available to eligible patients through state-funded drug provision programs. Biologic agents used in clinical practice include anti-TNF agents (infliximab, adalimumab and golimumab), vedolizumab, and ustekinumab, with access determined by approved indications and reimbursement criteria. Small-molecule therapies have also been registered in Kazakhstan; however, they are not yet routinely provided to patients with IBD through the state-funded reimbursement programs. Thus, access to advanced IBD therapy in routine practice remains partly determined by the national reimbursement framework. Advanced therapies are typically initiated and monitored in specialized gastroenterology and IBD centers.

Against this background, the establishment of a national multicenter clinical IBD registry became particularly important to systematically collect data from patients managed in different regions of Kazakhstan and to provide a more comprehensive characterization of disease patterns and clinical management. The aim of the present study was to evaluate the clinical and demographic characteristics of patients included in the national multicenter clinical IBD registry of the Republic of Kazakhstan according to disease activity at registry enrollment and to identify factors associated with active disease at that time.

## 2. Materials and Methods

### 2.1. Study Design and Setting

The present study represents a cross-sectional analysis of data obtained at the enrollment visit in the registry (visit 0), within the framework of the national multicenter clinical registry of IBD in the Republic of Kazakhstan. Although the registry itself is non-interventional, prospective, and longitudinal, the present study analyzed only the data obtained at the time of patient inclusion in the registry; data from subsequent longitudinal follow-up were not included in the analysis.

Specialized centers from various regions of Kazakhstan, providing medical care to patients with IBD at secondary and tertiary levels, participated in the IBD Registry. Enrollment was conducted both in inpatient and outpatient settings, with a significant proportion of patients being enrolled during hospitalization. Eligible patients were consecutively included in the registry upon presenting to the participating centers, either for an initial consultation or a scheduled follow-up visit, provided they met the inclusion criteria. In this context, the term “national” reflects the geographical coverage of the registry and the participation of specialized centers from different regions of Kazakhstan, rather than a population-based principle implying the identification of all IBD cases in the country.

### 2.2. Ethical Approval

The study was conducted in accordance with the principles of the World Medical Association’s Helsinki Declaration and the requirements of Good Clinical Practice (ICH-GCP). The study protocol was reviewed and approved by the local ethics committee of JSC “Asfendiyarov Kazakh National Medical University” (excerpt from the protocol of the LEC meeting No. 15(121) dated 24 November 2021). All study participants provided written informed consent to participate and to process personal data before being included in the registry.

### 2.3. Study Population

This analysis included adult patients with a confirmed diagnosis of ulcerative colitis or Crohn’s disease, registered in the IBD Registry between December 2021 and December 2024. The final analytical cohort included 1521 patients from 18 participating centers. The centers were distributed as follows: three centers in Shymkent and the Turkestan region; two centers each in Astana, Pavlodar, and Karaganda; and one center each in Almaty, the Akmola, North Kazakhstan, Kyzylorda, Aktobe, East Kazakhstan, Mangystau, Zhambyl, and Almaty regions. The number of patients enrolled at individual centers ranged from 11 to 417.

Patients with unclassified IBD (IBD-U) were not included in this analysis. For the present cross-sectional analysis, only data from the first visit to the registry (visit 0), corresponding to the time of patient inclusion in the study, were used. Data from subsequent longitudinal follow-up were not analyzed.

Disease activity at the enrollment visit was assessed using disease-specific clinical indices. For ulcerative colitis, the full Mayo score (range 0–12 points) was used. The endoscopic subscore of the full Mayo score was derived from the most recent colonoscopy findings available in the medical record at the time of registry enrollment. According to the threshold values applied in the present study, 0–2 points corresponded to remission, 3–5 points to mild, 6–10 points to moderate, and 11–12 points to severe disease activity. For the dichotomous analysis, Mayo 0–2 was defined as remission, and Mayo ≥ 3 as active disease.

Crohn’s disease activity was assessed using the Harvey–Bradshaw Index (HBI): 0–4 points corresponded to remission, 5–7 points to mild, 8–16 points to moderate, and ≥17 points to severe activity. For the dichotomous analysis, HBI 0–4 was defined as remission, and HBI ≥ 5 as active disease.

The frequency of exacerbations was recorded at the registry enrollment visit as a categorical variable using three predefined response options in the registry form: 0–1 exacerbation per year, ≥2 exacerbations per year, and 1 exacerbation every 2–3 years or less frequently. Only one response option could be selected for each patient. The treating physician assigned the category based on the patient’s clinical history and available medical documentation. The registry case report form did not specify a fixed retrospective look-back period for exacerbation frequency; classification was based on the available clinical history and medical documentation at the time of enrollment. The case report form also did not distinguish previous exacerbations from the exacerbation present at the enrollment visit; therefore, the current episode could have contributed to the assigned exacerbation-frequency category. Although the wording “0–1 exacerbation per year” may formally overlap numerically with “1 exacerbation every 2–3 years or less frequently,” these categories should be interpreted as separate predefined registry response options rather than strictly non-overlapping numerical intervals.

### 2.4. Eligibility Criteria

The inclusion and exclusion criteria are presented in Table 1.

### 2.5. Statistical Analysis

Statistical analysis was performed using the R software environment version 4.2.3 (R Foundation for Statistical Computing, Vienna, Austria). Quantitative variables are presented as median and interquartile range (IQR), while categorical variables are presented as absolute values and percentages. The Mann–Whitney test (Wilcoxon rank-sum test) was used to compare quantitative variables, and the Pearson χ^2^ test or Fisher’s exact test was used for categorical variables. For pooled analyses of UC and CD, disease activity was defined using the corresponding disease-specific indices (full Mayo score for UC and Harvey-Bradshaw Index for CD). Therefore, pooled comparisons should be interpreted descriptively, as they combine two different disease-specific definitions of activity and observed differences may partly reflect the underlying UC/CD case mix.

To identify factors associated with active disease at the time of registry enrollment visit (visit 0), separate univariable and multivariable logistic regression analyses were conducted for ulcerative colitis and Crohn’s disease. The results are presented as odds ratios (OR) and adjusted odds ratios (aOR) with 95% confidence intervals (CIs). For conventional logistic regression models, 95% confidence intervals were calculated using the Wald method, and *p*-values were based on Wald tests. For Firth penalized logistic regression sensitivity analyses, confidence intervals and *p*-values were obtained using penalized-likelihood procedures implemented in the logistf package (version 1.26.1; Georg Heinze, Medical University of Vienna, Vienna, Austria).

Covariates for multivariable models were predetermined based on their clinical significance, potential role as confounders, and availability of corresponding data in the database, regardless of statistical significance in univariable analysis. The model included age, sex, body mass index, place of residence, smoking status, disease duration, frequency of exacerbations, history of surgery related to IBD, current therapy (5-ASA, corticosteroids, immunosuppressive and biological therapy), disease extent or location according to the Montreal classification, intestinal complications, and extraintestinal manifestations (EIMs). Treatment categories were not mutually exclusive, as patients could receive concomitant therapies; therefore, percentages across treatment categories do not sum to 100%. Univariable analysis was used to describe unadjusted associations and was not applied as a criterion for including variables in multivariable models.

Multivariable analysis was conducted on observations with complete data. Model characteristics were assessed using the likelihood ratio test, Nagelkerke pseudo-R^2^, area under the ROC curve (AUC), and the Hosmer–Lemeshow goodness-of-fit test. Multicollinearity among covariates was evaluated using the generalized variance inflation factor (GVIF) adjusted for degrees of freedom.

Given the relatively limited number of observations in some covariate categories, sensitivity analyses using Firth penalized logistic regression were performed for both the ulcerative colitis and Crohn’s disease multivariable models. To assess whether the inclusion of extraintestinal manifestations in the multivariable model affected the estimates of other factors, the model for Crohn’s disease was recalculated after excluding EIMs from the covariates while retaining the other variables and the same analytical sample. To assess the potential impact of complete-case exclusion due to missing smoking-status data, the multivariable models for both ulcerative colitis and Crohn’s disease were also refitted after omitting smoking status as a covariate, thereby allowing inclusion of the full disease-specific cohorts. Differences were considered statistically significant at *p* < 0.05. No adjustment for multiple comparisons was performed, and the analyses should therefore be considered exploratory.

## 3. Results

**Figure 1:**
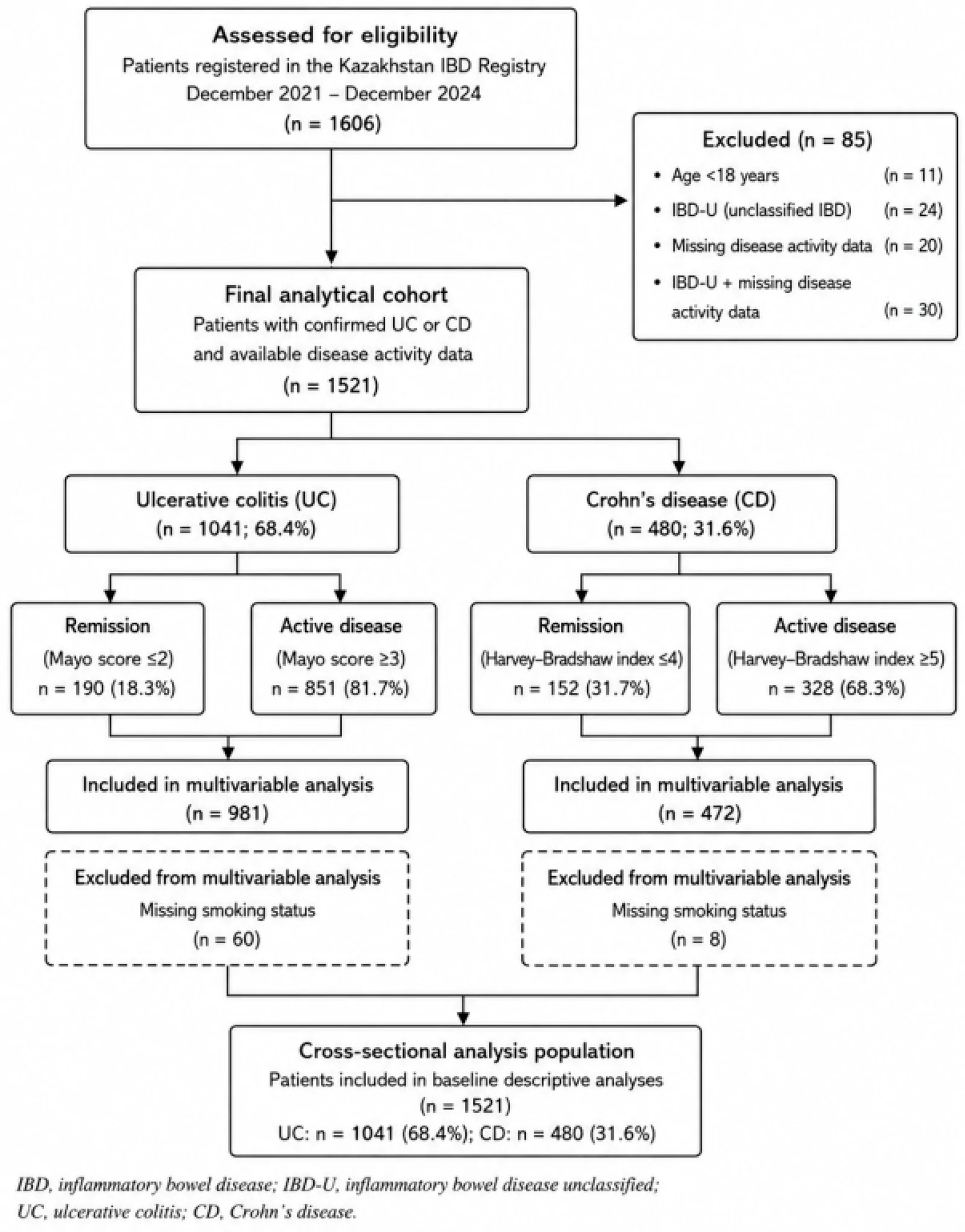
Participant flow diagram for the present cross-sectional analysis of the national multicenter IBD registry of the Republic of Kazakhstan.

The final study cohort consisted of 1521 patients, 1041 (68.4%) with ulcerative colitis (UC) and 480 (31.6%) with Crohn’s disease (CD). At the registry enrollment visit (visit 0), active disease was recorded in 1179 (77.5%) patients with IBD, while clinical remission was observed in 342 (22.5%) patients. The participant selection process is summarized in Figure 1.

Table 2 presents the characteristics of patients with IBD according to disease activity at the registry enrollment visit (visit 0), including demographic features, disease characteristics, and current treatment. Compared with patients in remission, patients with active disease were younger (median age 43.0 vs. 45.5 years; *p* = 0.008), had a slightly lower body mass index (23.0 vs. 23.4 kg/m^2^; *p* = 0.006), and more often lived in rural areas (20.4% vs. 12.6%; *p* = 0.001). Frequent exacerbations (≥2 per year) were also more common among patients with active disease (38.5% vs. 17.8%; *p* < 0.001), as were extraintestinal manifestations (23.9% vs. 14.3%; *p* < 0.001). Regarding current therapy, 93.3% of patients were receiving 5-ASA preparations, 24.5% corticosteroids, 21.5% biological therapy, and 9.6% immunosuppressive therapy. At the time of registry inclusion, 28.1% of patients with active disease and 12.3% of patients in remission were receiving corticosteroids (*p* < 0.001). The frequency of biological therapy use did not differ significantly between groups (21.2% vs. 22.5%; *p* = 0.604).

### 3.1. Ulcerative Colitis

Among patients with UC (Table 3), active disease was observed in 851 of 1041 patients (81.7%), while 190 (18.3%) were in remission. Rural residence was significantly more common among patients with active UC compared to those in remission (22.4% vs. 11.1%; *p* < 0.001). Patients with active UC also more frequently had ≥2 flares per year (36.9% vs. 13.7%; *p* < 0.001) and extraintestinal manifestations (19.6% vs. 13.2%; *p* = 0.038). The prevalence of disease according to the Montreal classification did not differ statistically significantly between activity groups (*p* = 0.804).

In the univariable analysis (Table 4), active UC was associated with living in rural areas (OR 2.33; 95% CI 1.44–3.77; *p* < 0.001), having ≥2 flares per year (OR 3.36; 95% CI 2.16–5.23; *p* < 0.001), use of corticosteroids (OR 6.95; 95% CI 3.61–13.36; *p* < 0.001), immunosuppressive therapy (OR 2.42; 95% CI 1.09–5.34; *p* = 0.029), and the presence of extraintestinal manifestations (OR 1.61; 95% CI 1.02–2.54; *p* = 0.039). Rare flares (≤1 time in 2–3 years) were associated with lower odds of active disease (OR 0.46; 95% CI 0.28–0.78; *p* = 0.003). In the multivariable model (Table 4), a statistically significant association with active UC remained for rural residence (adjusted OR 2.11; 95% CI 1.25–3.56; *p* = 0.005), ≥2 flares per year (OR 3.47; 95% CI 2.18–5.51; *p* < 0.001), use of corticosteroids (OR 5.96; 95% CI 3.04–11.69; *p* < 0.001), and immunosuppressive therapy (OR 2.71; 95% CI 1.15–6.38; *p* = 0.022). Rare exacerbations remained inversely associated with active disease (adjusted OR 0.41; 95% CI 0.23–0.73; *p* = 0.002). After adjustment, extraintestinal manifestations lost statistical significance (*p* = 0.298). Associations with corticosteroid and immunosuppressive therapy should be interpreted in light of the cross-sectional study design, as the prescription of these drugs could have been a consequence of higher disease activity rather than a factor preceding its development.

Given the small number of exposed patients in several covariate categories, the multivariable model for ulcerative colitis was refitted using Firth penalized logistic regression. The principal findings were materially unchanged. Rural residence remained associated with higher odds of active disease (Firth OR 2.05, 95% CI 1.25–3.50; *p* = 0.004), as did frequent exacerbations (≥2/year; OR 3.35, 95% CI 2.15–5.39; *p* < 0.001), corticosteroid therapy (OR 5.56, 95% CI 3.02–11.35; *p* < 0.001), and immunosuppressive therapy (OR 2.53, 95% CI 1.17–6.24; *p* = 0.017). Exacerbations occurring 1 every 2–3 years or less frequently remained inversely associated with active disease (OR 0.42, 95% CI 0.24–0.74; *p* = 0.003). The estimate for intestinal complications was attenuated and remained imprecise and statistically non-significant (OR 2.32, 95% CI 0.73–9.77; *p* = 0.164) (Supplementary Table S1, Panel A).

### 3.2. Crohn’s Disease

In the CD subgroup (Table 5), active disease was observed in 328 out of 480 patients (68.3%). Several clinical characteristics and disease features differed depending on disease activity. In patients with active Crohn’s disease, the median BMI was lower than in patients in remission (22.7 vs. 23.2 kg/m^2^; *p* = 0.021). Differences were also observed depending on disease location. Ileocolitis (L3) was present in half of the patients with active CD (50.0% vs. 42.1% in remission) whereas among patients in remission, isolated involvement of the terminal ileum (L1) predominated (25.0% vs. 15.9%; *p* = 0.019). Active disease was also associated with a higher frequency of exacerbations: ≥2 exacerbations per year were reported in 42.7% of patients with active disease compared with 23.0% of those in remission (*p* < 0.001). Extraintestinal manifestations were substantially more common in patients with active CD (35.1% vs. 15.8%; *p* < 0.001). Overall, intestinal complications were recorded in 7.7% of patients with CD, while a history of IBD-related surgery was noted in 24.0% of patients.

In the multivariable analysis (Table 6), several factors remained independently associated with active Crohn’s disease. Frequent flares (≥2 times per year) were associated with higher odds of active disease (adjusted OR 2.41; 95% CI 1.47–3.95; *p* < 0.001), whereas infrequent flares (≤1 time in 2–3 years) were associated with significantly lower odds of disease activity (adjusted OR 0.24; 95% CI 0.09–0.61; *p* = 0.003). Compared with isolated ileal involvement (L1), both colonic involvement (L2) (adjusted OR 2.32; 95% CI 1.25–4.30; *p* = 0.008) and ileocolitis (L3) (adjusted OR 2.24; 95% CI 1.25–4.04; *p* = 0.007) were independently associated with active disease. One of the most pronounced associations with disease activity was the presence of extraintestinal manifestations (adjusted OR 2.88; 95% CI 1.68–4.93; *p* < 0.001). Corticosteroid therapy was also associated with higher odds of active disease (adjusted OR 1.77; 95% CI 1.03–3.05; *p* = 0.039), whereas immunosuppressive therapy was associated with lower odds of active disease (adjusted OR 0.46; 95% CI 0.24–0.89; *p* = 0.021).

At the same time, BMI and rural residence, after adjustment, were not independently associated with Crohn’s disease activity (adjusted OR 0.97; 95% CI 0.92–1.01; *p* = 0.172 and adjusted OR 0.88; 95% CI 0.48–1.61; *p* = 0.671, respectively). Biological therapy was also not associated with disease activity in the adjusted model (adjusted OR 0.99; 95% CI 0.57–1.70; *p* = 0.966). Consistent with these results, the proportion of patients receiving biological therapy was comparable in the remission and active disease groups (26.3% versus 29.0%), as was the frequency of immunosuppressive therapy use (15.8% versus 13.1%).

The multivariable logistic regression model for Crohn’s disease included 472 patients with complete data, of whom 322 had active disease and 150 were in remission. The overall model was statistically significant compared with the intercept-only model (likelihood-ratio χ^2^(18) = 79.89, *p* < 0.001), with a Nagelkerke pseudo-R^2^ of 0.218. The model demonstrated acceptable discrimination (AUC = 0.735, 95% CI 0.687–0.783). The Hosmer–Lemeshow test showed no significant evidence of lack of fit (χ^2^(8) = 12.29, *p* = 0.139). No problematic multicollinearity was detected (adjusted GVIF range 1.014–1.131). The ratio of observations in the less frequent outcome category to estimated predictor parameters was 8.3, indicating a relatively limited events-per-parameter margin.

Given the relatively limited number of remission cases relative to the number of estimated parameters in the Crohn’s disease model (150 remission cases; 18 parameters), Firth penalized logistic regression was performed as a sensitivity analysis. The Firth estimates were highly consistent with the conventional maximum-likelihood estimates, with no changes in the direction or statistical significance of the principal associations (Supplementary Table S1, Panel B).

To assess the robustness of the multivariable model to potential overlap between extraintestinal manifestations (EIMs) and the complications component of the Harvey–Bradshaw Index, the model was refitted after excluding EIMs as a predictor. The principal associations remained materially unchanged. Frequent exacerbations (≥2/year) remained associated with higher odds of active disease (aOR 2.42, 95% CI 1.49–3.93; *p* < 0.001), whereas exacerbations occurring 1 every 2–3 years or less frequently remained inversely associated with active disease (aOR 0.26, 95% CI 0.11–0.64; *p* = 0.003). The associations with corticosteroid therapy (aOR 1.95, 95% CI 1.15–3.32; *p* = 0.013), L2 disease location (aOR 2.30, 95% CI 1.25–4.21; *p* = 0.007), and L3 disease location (aOR 2.31, 95% CI 1.30–4.12; *p* = 0.004) were also preserved. The inverse association with immunosuppressive therapy was attenuated and no longer reached statistical significance (aOR 0.55, 95% CI 0.29–1.05; *p* = 0.069). Overall, exclusion of EIMs did not materially alter the principal effect estimates, supporting the robustness of the main findings (Supplementary Table S2).

To assess the potential impact of complete-case exclusion due to missing smoking-status data, the multivariable models were refitted after omitting smoking status as a covariate. This allowed inclusion of the full disease-specific cohorts (UC, n = 1041; CD, n = 480), compared with 981 and 472 patients, respectively, in the primary models. The principal associations were materially unchanged in both UC and CD, with no changes in the direction or statistical significance of the main associations identified in the primary analyses (Supplementary Table S3, Panels A and B).

**Figure 2:**
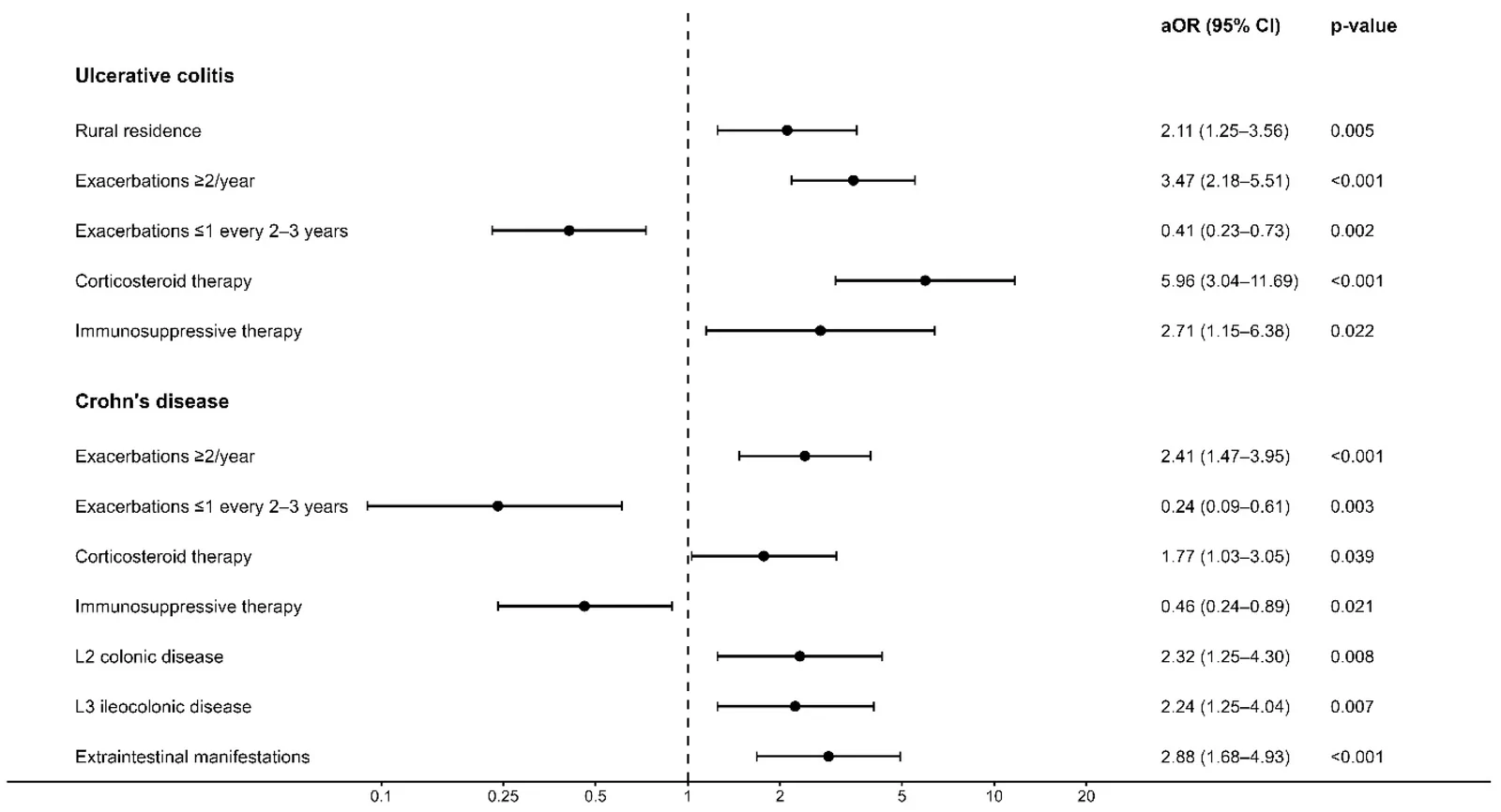
Adjusted odds ratios for factors associated with active ulcerative colitis and Crohn’s disease at the registry enrollment visit (visit 0).

The statistically significant adjusted associations from the primary UC and CD multivariable models are summarized in Figure 2.

Only predictors that were statistically significantly associated with active disease (*p* < 0.05) in the respective multivariable logistic regression models are displayed. Points represent adjusted odds ratios (aORs), and horizontal bars represent Wald 95% confidence intervals. The vertical dashed line indicates the null value (aOR = 1.0). Complete results for all covariates are presented in Table 4 and Table 6.

## 4. Discussion

To our knowledge, this study is the first large multicenter registry-based analysis describing the clinical activity of IBD, disease phenotypes, and treatment patterns in Kazakhstan—a region that remains underrepresented in international IBD data. The main findings were a high proportion of clinically active disease at the registry enrollment visit (visit 0) (77.5%), distinct patterns of factors associated with activity in UC and CD, and no significant differences in the frequency of biological therapy between patients in remission and those with active disease. In UC, activity was independently associated with rural residence, frequent exacerbations, and current use of corticosteroids and immunosuppressants. In CD, activity was independently associated with frequent exacerbations, colonic or ileocolonic disease location, extraintestinal manifestations, and corticosteroid therapy, whereas immunosuppressive therapy was associated with lower odds of active disease. These results should be interpreted as cross-sectional associations rather than causal factors or prognostic predictors.

The high proportion of patients with active disease is a clinically significant sign, but it should not be considered as the population prevalence of uncontrolled IBD in Kazakhstan. The registry was mainly formed in secondary and tertiary centers and included patients at the time they sought specialized care, which could have led to a predominance of more symptomatic and complex cases. In addition, activity was determined using the full Mayo and Harvey–Bradshaw indexes, whereas modern treat-to-target strategies involve combining symptom assessment with biochemical, endoscopic, and, for Crohn’s disease, transmural markers [13]. Therefore, the observed proportion of 77.5% reflects the clinical status of the cohort at the registry enrollment visit, but it does not allow us to conclude about the frequency of objectively active inflammation or failure to reach endoscopic targets across the entire IBD population in Kazakhstan.

The phenotypic distribution was generally comparable to that reported in international cohorts: in most patients with UC, inflammation extended beyond the rectum, while in CD, the most common location was ileocolitis [14,15,16]. The predominance of inflammatory behavior in CD may indicate a significant portion of patients without recorded stricturing or penetrating complications at the time of inclusion. The median duration of CD was six years, and 24.0% of patients had a history of surgical interventions related to IBD. At the same time, intestinal complications at the time of enrollment were recorded in only 7.7% of patients. This discrepancy likely reflects differences in the timeframes of the evaluated indicators: surgical history characterizes events that occurred throughout the entire previous course of the disease, whereas the presence of complications was assessed at the time of registry enrollment.

An independent association between rural living and active UC was one of the notable findings of the study. Patients living in rural areas had approximately twice the likelihood of active ulcerative colitis compared to urban residents after accounting for other factors (aOR 2.11; 95% CI 1.25–3.56). Possible explanations could be the distance to specialized centers, limited access to endoscopic and lab monitoring, referral delays, and differences in continuity of care; similar geographical and socio-economic disparities have been described in previous systematic reviews on IBD [17]. However, the registry did not assess distance to healthcare facilities, income, education, insurance coverage, consultation timing, or availability of specific medications. Moreover, living in a rural area was not associated with Crohn’s disease activity in the adjusted model (aOR 0.88; 95% CI 0.48–1.61). Therefore, living in a rural area should be considered a potential marker of barriers to healthcare access, warranting further investigation, rather than a direct cause of ulcerative colitis activity.

Unlike ulcerative colitis, Crohn’s disease activity was mainly associated with phenotypic characteristics of the disease. In Crohn’s disease, the localization of the disease and extraintestinal manifestations proved to be particularly important. Compared with isolated involvement of the ileum (L1), colonic localization (L2) and ileocolonic localization (L3) were independently associated with approximately a twofold increase in the likelihood of active disease (aOR 2.32 and 2.24, respectively). Extraintestinal manifestations demonstrated one of the strongest associations with active Crohn’s disease (aOR 2.88; 95% CI 1.68–4.93): they were observed in 35.1% of patients with active disease compared with 15.8% of patients in remission. These results are consistent with the concept of Crohn’s disease as a heterogeneous systemic immune-mediated disorder, in which the disease phenotype and extra-gastrointestinal manifestations can largely determine the overall clinical burden [18,19]. At the same time, this association should be interpreted with some caution, since extraintestinal complications are included in the structure of the Harvey–Bradshaw index used to assess disease activity. In further studies, the use of objective markers of inflammation or activity definitions independent of extraintestinal manifestations could help to more accurately evaluate this relationship.

Patients with active CD also had a slightly lower median BMI than those in remission (22.7 vs. 23.2 kg/m^2^), and BMI was associated with activity in the univariable analysis. However, this association was no longer statistically significant after multivariable adjustment (aOR 0.97 per 1 kg/m^2^ increase; 95% CI 0.92–1.01; *p* = 0.172). Therefore, in contrast to the initial unadjusted findings, lower BMI cannot be considered independently associated with active CD in the present analysis. The observed crude difference may still reflect greater inflammatory burden, reduced nutritional intake, malabsorption, or other clinical and socioeconomic factors accompanying active disease, but the current data do not demonstrate an independent relationship.

Frequent exacerbations were one of the most consistent factors associated with active disease in both UC and CD. In UC, ≥2 exacerbations per year were associated with more than a threefold increase in the odds of active disease (aOR 3.47; 95% CI 2.18–5.51), whereas exacerbations occurring ≤1 time in 2–3 years were associated with lower odds of activity (aOR 0.41; 95% CI 0.23–0.73). A similar pattern was observed in CD, although the magnitude of the association was somewhat lower: ≥2 exacerbations per year were associated with higher odds of active disease (aOR 2.41; 95% CI 1.47–3.95), whereas rare exacerbations were associated with significantly lower odds of activity (aOR 0.24; 95% CI 0.09–0.61). Although this result is clinically expected, it emphasizes that recurrent exacerbations allow for the identification of a subgroup of patients with persistent inadequate disease control and may serve as a useful marker for closer monitoring and reassessment of therapeutic strategies.

The therapy structure requires especially careful interpretation. The use of 5-ASA was predictably high in UC (97.2%), but it was also noted in 84.8% of patients with CD. Current guidelines do not generally support 5-ASA as an effective therapy for active luminal CD, except in certain limited clinical circumstances [20]. However, indications, doses, treatment duration, and previous treatment regimens were not evaluated in the present cross-sectional analysis; therefore, these data do not allow assessment of the appropriateness of 5-ASA use in individual patients.

Corticosteroid therapy was strongly associated with active UC (aOR 5.96; 95% CI 3.04–11.69) and was also independently associated with active CD after adjustment (aOR 1.77; 95% CI 1.03–3.05). These associations should not be interpreted as evidence that corticosteroid exposure increases the risk of active disease. In a cross-sectional analysis, corticosteroids are commonly prescribed because a patient is experiencing an active flare; therefore, reverse causation and confounding by indication are highly plausible. Accordingly, the observed associations are more likely to reflect treatment of ongoing disease activity at the time of registry inclusion rather than an independent adverse effect of corticosteroid therapy.

The results regarding immunosuppressive therapy differed between UC and CD. In UC, it was associated with higher odds of active disease (aOR 2.71; 95% CI 1.15–6.38), whereas in CD it was associated with lower odds of active disease in the primary multivariable model (aOR 0.46; 95% CI 0.24–0.89). In CD, this inverse association remained statistically significant in the Firth penalized sensitivity analysis (aOR 0.47; 95% CI 0.25–0.91), but was attenuated and no longer reached conventional statistical significance when EIMs were excluded from the model (aOR 0.55; 95% CI 0.29–1.05; *p* = 0.069). Thus, although the direction of the association was consistent across analyses, its statistical robustness was limited and the finding should be interpreted cautiously. The opposite directions observed in UC and CD are most plausibly explained by differential treatment channeling, including differences in disease phenotype, severity, and treatment history, rather than by a disease-specific pharmacological effect.

The comparable frequency of biological therapy between patients with active disease and those in remission is another important finding. In the overall cohort, biologic therapy was used in 21.2% of patients with active disease and 22.5% of those in remission. Similarly, no significant differences were observed within UC (18.2% vs. 19.5%) or CD (29.0% vs. 26.3%). Although biologic therapies have demonstrated effectiveness in achieving treatment targets in IBD cohorts treated in routine clinical practice [21], biologic therapy was not independently associated with disease activity in our analysis. This finding should not be interpreted as evidence of insufficient treatment effectiveness, as the cross-sectional design does not allow assessment of treatment response over time and is susceptible to confounding by indication and reverse causation.

The strengths of the study include a large sample size, multicenter coverage of regions in Kazakhstan, standardized registry-based description of phenotypes, and separate analysis of UC and CD. The study provides rare registry-based clinical data from Central Asia and creates a basis for assessing geographic differences, treatment dynamics, and clinical outcomes. The multivariable models also allowed the identification of disease-specific association patterns, while additional sensitivity analyses in the CD subgroup generally supported the main findings, although the statistical significance of some individual associations varied across model specifications. At the same time, the cross-sectional nature of the present analysis, referral of many patients to specialized centers, the fact that detailed longitudinal treatment data were not included in the present analysis, and reliance primarily on clinical activity indices limit causal and prognostic interpretation. Future longitudinal and center-stratified analyses incorporating biochemical, endoscopic, and imaging outcomes are needed to determine whether the observed associations translate into differences in sustained disease control and long-term outcomes.

## 5. Limitations

The study has several limitations. First, the analysis was limited to data from the registry enrollment visit and was therefore cross-sectional, precluding assessment of temporal relationships or causal associations. This is particularly important when interpreting associations between disease activity and current therapy, which may be affected by reverse causation and confounding by indication. Because exacerbation frequency was recorded at the registry enrollment visit and disease activity was assessed at the same time point, the temporal relationship between these variables cannot be established. Moreover, the registry did not distinguish previous exacerbations from the episode present at enrollment, and the current exacerbation could therefore have contributed to the assigned frequency category. Accordingly, the strong association between frequent exacerbations and active disease should be interpreted as cross-sectional rather than predictive or causal.

Second, the predominance of patients enrolled in the registry at secondary- and tertiary-level institutions, especially during hospitalization, may have resulted in referral and selection bias toward patients with more active or complicated disease. Therefore, the observed frequency of active disease and therapy characteristics may not be fully generalizable to the entire population of IBD patients in Kazakhstan, particularly to patients with a milder disease course who are managed exclusively in outpatient settings.

Third, differences between participating centers in data completeness, assessment of disease activity, and therapeutic approaches cannot be excluded; moreover, clustering by center was not accounted for in the regression models. A formal center-level clustered or multilevel analysis could not be performed because individual-level center identifiers were not available for all participants in the final analytical dataset. Therefore, residual within-center correlation cannot be excluded and may have affected the precision of the estimated associations. Objective markers of inflammatory activity, including fecal calprotectin, C-reactive protein, endoscopic activity, and small bowel imaging, were also not standardized across the entire cohort.

In addition, because EIMs may overlap with the complications component of the HBI, the association between EIMs and HBI-defined Crohn’s disease activity may be affected by incorporation bias. Although sensitivity analysis excluding EIMs from the multivariable model demonstrated that the principal associations with other covariates were robust, a modified HBI excluding overlapping EIM-related components could not be reconstructed because item-level HBI complication data were unavailable. Therefore, the observed association between EIMs and disease activity should be interpreted with caution.

Fourth, detailed characteristics of therapy, including its duration, dosing, sequence, and temporal relationship with disease activity, were not included in this cross-sectional analysis. Therefore, the identified associations between current therapy and disease activity should not be interpreted as an assessment of treatment efficacy.

Finally, despite the longitudinal nature of the registry, subsequent follow-up, including hospitalizations, surgical interventions, therapy changes, and achievement of clinical or objective remission, was not within the scope of this analysis. Therefore, the results obtained should be interpreted as associations identified at the time of registry inclusion, rather than as causal or prognostic relationships.

## 6. Conclusions

In this multicenter clinical IBD registry cohort from Kazakhstan, most patients had clinically active disease at the time of registry enrollment. The factors associated with activity differed between UC and CD. In UC, rural residence and frequent exacerbations were independently associated with active disease, whereas in CD, frequent exacerbations, colonic or ileocolonic location, and extraintestinal manifestations were the principal clinical factors associated with active disease. Biologic therapy was used at comparable frequencies in patients with active disease and those in remission and was not independently associated with activity in either UC or CD. Treatment-related associations should be interpreted cautiously because of the cross-sectional design and potential confounding by indication. Longitudinal analysis of registry follow-up data will allow further evaluation of treatment trajectories, achievement of clinical and objective remission, and subsequent clinical outcomes. The present findings characterize disease activity and its associated factors at registry inclusion but do not support causal conclusions.

## Figures and Tables

**Table 1:** Inclusion/exclusion criteria.

Inclusion Criteria	Exclusion Criteria
Age ≥ 18 years	Age < 18 years
A confirmed diagnosis of ulcerative colitis or Crohn’s disease based on endoscopic, histological, and/or imaging methods	Taking part in an interventional clinical trial
Enrollment in the IBD Registry of the Republic of Kazakhstan between December 2021 and December 2024	Unconfirmed diagnosis of ulcerative colitis or Crohn’s disease at the registry enrollment visit (visit 0)
A signed informed consent to participate in the registry	Missing baseline data required for disease activity classification
Citizenship of the Republic of Kazakhstan or permanent residence in the territory of the Republic of Kazakhstan	IBD-unclassified
Availability of disease activity data at visit 0	

**Table 2:** Characteristics of patients with IBD according to disease activity at the registry enrollment visit (visit 0).

Variable	Overall (N = 1521) ^1^	Remission (*n* = 342) ^1^	Active Disease (n = 1179) ^1^	*p*-Value ^2^
Demographic characteristics				
Age, years	43.0 (34.0–56.0)	45.5 (35.0–59.0)	43.0 (34.0–55.0)	0.008
Sex, male	748 (49.2%)	151 (44.2%)	597 (50.6%)	0.035
BMI, kg/m^2^	23.1 (20.1–26.1)	23.4 (20.7–26.8)	23.0 (20.0–26.0)	0.006
Residence, rural	283 (18.6%)	43 (12.6%)	240 (20.4%)	0.001
Smoking status				0.678
Yes	143 (9.4%)	31 (9.1%)	112 (9.5%)	
No	1310 (86.1%)	307 (89.8%)	1003 (85.1%)	
Missing data ^3^	68 (4.5%)	4 (1.2%)	64 (5.4%)	
Disease characteristics				
Diagnosis				<0.001
Ulcerative colitis	1041 (68.4%)	190 (55.6%)	851 (72.2%)	
Crohn’s disease	480 (31.6%)	152 (44.4%)	328 (27.8%)	
Disease duration, years	5.0 (3.0–10.0)	6.0 (3.0–10.0)	5.0 (3.0–10.0)	0.289
Exacerbation frequency				<0.001
0–1/year	905 (59.5%)	235 (68.7%)	670 (56.8%)	
≥2/year	515 (33.9%)	61 (17.8%)	454 (38.5%)	
1 every 2–3 years or less frequently	101 (6.6%)	46 (13.5%)	55 (4.7%)	
Previous IBD-related surgery, yes	160 (10.5%)	48 (14.0%)	112 (9.5%)	0.016
Intestinal complications				0.283
None	1447 (95.1%)	332 (97.1%)	1115 (94.6%)	
Abscess	11 (0.7%)	1 (0.3%)	10 (0.8%)	
GI bleeding	28 (1.8%)	2 (0.6%)	26 (2.2%)	
Obstruction	21 (1.4%)	4 (1.2%)	17 (1.4%)	
Perforation	11 (0.7%)	3 (0.9%)	8 (0.7%)	
Toxic megacolon	3 (0.2%)	0 (0.0%)	3 (0.3%)	
Extraintestinal manifestations, yes	331 (21.8%)	49 (14.3%)	282 (23.9%)	<0.001
Current treatment ^4^				
5-ASA therapy, yes	1419 (93.3%)	305 (89.2%)	1114 (94.5%)	<0.001
Corticosteroid therapy, yes	373 (24.5%)	42 (12.3%)	331 (28.1%)	<0.001
Immunosuppressive therapy, yes	146 (9.6%)	31 (9.1%)	115 (9.8%)	0.703
Biologic therapy, yes	327 (21.5%)	77 (22.5%)	250 (21.2%)	0.604

^1^ Median (25–75%); n (%); ^2^ Wilcoxon rank-sum test; Pearson’s χ^2^ test or Fisher’s exact test, as appropriate. ^3^ Missing values are presented descriptively but were excluded from statistical comparisons. ^4^ Treatment categories were not mutually exclusive; patients could receive more than one concomitant therapy.

**Table 3:** Clinical characteristics and current treatment of patients with ulcerative colitis according to disease activity at the registry enrollment visit (visit 0).

Variable	Overall ^1^	Remission ^1^	Active Disease ^1^	*p*-Value ^2^
Demographic characteristics				
Age, years	43.0 (34.0–56.0)	45.0 (35.0–58.0)	43.0 (34.0–55.0)	0.112
Sex				0.184
Male	522 (50.1%)	87 (45.8%)	435 (51.1%)	
Female	519 (49.9%)	103 (54.2%)	416 (48.9%)	
BMI, kg/m^2^	23.4 (20.1–26.2)	23.8 (20.6–27.0)	23.3 (20.0–26.1)	0.052
Residence				<0.001
Urban	829 (79.6%)	169 (88.9%)	660 (77.6%)	
Rural	212 (20.4%)	21 (11.1%)	191 (22.4%)	
Smoking status				0.467
Yes	83 (8.0%)	13 (6.8%)	70 (8.2%)	
No	898 (86.3%)	175 (92.1%)	723 (85.0%)	
Missing data ^3^	60 (5.8%)	2 (1.1%)	58 (6.8%)	
Disease characteristics				
Disease extent (Montreal classification)				0.804
E1 (Proctitis)	186 (17.9%)	33 (17.4%)	153 (18.0%)	
E2 (Left-sided colitis)	449 (43.1%)	86 (45.3%)	363 (42.7%)	
E3 (Extensive colitis)	406 (39.0%)	71 (37.4%)	335 (39.4%)	
Disease duration, years	5.0 (3.0–9.0)	6.0 (3.0–10.0)	5.0 (3.0–9.0)	0.176
Exacerbation frequency				<0.001
0–1/year	629 (60.4%)	137 (72.1%)	492 (57.8%)	
≥2/year	340 (32.7%)	26 (13.7%)	314 (36.9%)	
1 every 2–3 years or less frequently	72 (6.9%)	27 (14.2%)	45 (5.3%)	
Previous IBD-related surgery	45 (4.3%)	10 (5.3%)	35 (4.1%)	0.481
Intestinal complications				0.504
None	1004 (96.4%)	187 (98.4%)	817 (96.0%)	
Abscess	3 (0.3%)	1 (0.5%)	2 (0.2%)	
Gastrointestinal bleeding	25 (2.4%)	2 (1.1%)	23 (2.7%)	
Obstruction	3 (0.3%)	0 (0.0%)	3 (0.4%)	
Perforation	3 (0.3%)	0 (0.0%)	3 (0.4%)	
Toxic megacolon	3 (0.3%)	0 (0.0%)	3 (0.4%)	
Extraintestinal manifestations	192 (18.4%)	25 (13.2%)	167 (19.6%)	0.038
Current treatment ^4^				
5-ASA therapy	1012 (97.2%)	184 (96.8%)	828 (97.3%)	0.730
Corticosteroid therapy	247 (23.7%)	10 (5.3%)	237 (27.8%)	<0.001
Immunosuppressive therapy	79 (7.6%)	7 (3.7%)	72 (8.5%)	0.025
Biologic therapy	192 (18.4%)	37 (19.5%)	155 (18.2%)	0.686

^1^ Median (25–75%); n (%); ^2^ Wilcoxon rank-sum test; Pearson’s χ^2^ test or Fisher’s exact test, as appropriate. ^3^ Missing values are presented descriptively but were excluded from statistical comparisons. ^4^ Treatment categories were not mutually exclusive; patients could receive more than one concomitant therapy.

**Table 4:** Factors associated with active ulcerative colitis: univariable and multivariable logistic regression analyses.

Predictor	Crude OR (95% CI)	*p*-Value	Adjusted OR (95% CI)	*p*-Value
Age, per 1-year increase	0.99 (0.98–1.00)	0.113	1.00 (0.99–1.01)	0.841
BMI, per 1 kg/m^2^ increase	0.97 (0.94–1.01)	0.100	0.97 (0.93–1.01)	0.190
Disease duration, per 1-year increase	0.98 (0.97–1.00)	0.091	0.99 (0.97–1.01)	0.474
Female sex (vs. male)	0.81 (0.59–1.11)	0.185	0.87 (0.61–1.25)	0.450
Rural residence (vs. urban)	2.33 (1.44–3.77)	<0.001	2.11 (1.25–3.56)	0.005
Smoking (yes vs. no)	1.30 (0.70–2.41)	0.398	1.33 (0.68–2.60)	0.404
Exacerbation frequency ≥ 2/year	3.36 (2.16–5.23)	<0.001	3.47 (2.18–5.51)	<0.001
Exacerbation frequency 1 every 2–3 years or less frequently	0.46 (0.28–0.78)	0.003	0.41 (0.23–0.73)	0.002
Previous IBD-related surgery	0.77 (0.38–1.59)	0.482	0.74 (0.31–1.77)	0.492
5-ASA therapy	1.17 (0.47–2.92)	0.731	1.21 (0.45–3.27)	0.706
Corticosteroid therapy	6.95 (3.61–13.36)	<0.001	5.96 (3.04–11.69)	<0.001
Immunosuppressive therapy	2.42 (1.09–5.34)	0.029	2.71 (1.15–6.38)	0.022
Biologic therapy	0.92 (0.62–1.37)	0.686	0.73 (0.46–1.16)	0.188
E1 vs. E2	1.10 (0.70–1.71)	0.678	1.48 (0.91–2.41)	0.111
E3 vs. E2	1.12 (0.79–1.58)	0.530	1.01 (0.69–1.48)	0.972
Any intestinal complication	2.59 (0.79–8.53)	0.117	2.70 (0.70–10.46)	0.151
Extraintestinal manifestations	1.61 (1.02–2.54)	0.039	1.31 (0.79–2.15)	0.298

The multivariable logistic regression model included 981 patients with complete data; 60 observations were excluded because of missing data on smoking status. Of the 981 patients included in the model, 793 had active disease and 188 were in remission. The model was statistically significant compared with the intercept-only model (likelihood-ratio χ^2^(17) = 130.59, *p* < 0.001), with a Nagelkerke pseudo-R^2^ of 0.200. The model demonstrated acceptable discrimination (AUC = 0.755, 95% CI 0.718–0.792). The Hosmer–Lemeshow test showed no significant evidence of lack of fit (χ^2^(8) = 12.76, *p* = 0.120). No problematic multicollinearity was detected (adjusted GVIF range 1.008–1.108). The ratio of observations in the less frequent outcome category to estimated predictor parameters was 11.1.

**Table 5:** Clinical characteristics and current treatment of patients with Crohn’s disease according to disease activity at the registry enrollment visit (visit 0).

Variable	Overall ^1^	Remission ^1^	Active Disease ^1^	*p*-Value ^2^
Demographic characteristics				
Age, years	44.0 (34.0–57.0)	47.0 (35.0–64.0)	43.0 (33.0–55.0)	0.038
Sex				0.137
Male	226 (47.1%)	64 (42.1%)	162 (49.4%)	
Female	254 (52.9%)	88 (57.9%)	166 (50.6%)	
BMI, kg/m^2^	22.8 (20.1–26.0)	23.2 (20.7–26.6)	22.7 (19.7–25.8)	0.021
Residence				0.894
Urban	409 (85.2%)	130 (85.5%)	279 (85.1%)	
Rural	71 (14.8%)	22 (14.5%)	49 (14.9%)	
Smoking status				0.882
Yes	60 (12.5%)	18 (11.8%)	42 (12.8%)	
No	412 (85.8%)	132 (86.8%)	280 (85.4%)	
Missing data ^3^	8 (1.7%)	2 (1.3%)	6 (1.8%)	
Disease characteristics				
Disease location (Montreal classification)				0.019
L1 (Ileal disease)	90 (18.8%)	38 (25.0%)	52 (15.9%)	
L2 (Colonic disease)	154 (32.1%)	45 (29.6%)	109 (33.2%)	
L3 (Ileocolonic disease)	228 (47.5%)	64 (42.1%)	164 (50.0%)	
L4 (Upper GI disease)	8 (1.7%)	5 (3.3%)	3 (0.9%)	
Disease duration, years	6.0 (3.0–11.0)	6.0 (3.0–10.2)	6.0 (3.0–11.0)	0.518
Exacerbation frequency				<0.001
0–1/year	276 (57.5%)	98 (64.5%)	178 (54.3%)	
≥2/year	175 (36.5%)	35 (23.0%)	140 (42.7%)	
1 every 2–3 years or less frequently	29 (6.0%)	19 (12.5%)	10 (3.0%)	
Previous IBD-related surgery	115 (24.0%)	38 (25.0%)	77 (23.5%)	0.716
Intestinal complications				0.185
None	443 (92.3%)	145 (95.4%)	298 (90.9%)	
Abscess	8 (1.7%)	0 (0.0%)	8 (2.4%)	
GI bleeding	3 (0.6%)	0 (0.0%)	3 (0.9%)	
Obstruction	18 (3.8%)	4 (2.6%)	14 (4.3%)	
Perforation	8 (1.7%)	3 (2.0%)	5 (1.5%)	
Toxic megacolon	0 (0.0%)	0 (0.0%)	0 (0.0%)	
Extraintestinal manifestations	139 (29.0%)	24 (15.8%)	115 (35.1%)	<0.001
Current treatment ^4^				
5-ASA therapy	407 (84.8%)	121 (79.6%)	286 (87.2%)	0.031
Corticosteroid therapy	126 (26.2%)	32 (21.1%)	94 (28.7%)	0.078
Immunosuppressive therapy	67 (14.0%)	24 (15.8%)	43 (13.1%)	0.431
Biologic therapy	135 (28.1%)	40 (26.3%)	95 (29.0%)	0.548

^1^ Median (25–75%); n (%); ^2^ Wilcoxon rank-sum test; Pearson’s χ^2^ test or Fisher’s exact test, as appropriate. ^3^ Missing values are presented descriptively but were excluded from statistical comparisons. ^4^ Treatment categories were not mutually exclusive; patients could receive more than one concomitant therapy.

**Table 6:** Factors associated with active Crohn’s disease: univariable and multivariable logistic regression analyses.

Predictor	Crude OR (95% CI)	*p*-Value	Adjusted OR (95% CI)	Adjusted *p*
Age, per 1-year increase	0.99 (0.97–1.00)	0.019	0.99 (0.97–1.00)	0.136
BMI, per 1 kg/m^2^ increase	0.96 (0.92–1.00)	0.030	0.97 (0.92–1.01)	0.172
Disease duration, per 1-year increase	1.00 (0.98–1.02)	0.983	1.00 (0.98–1.02)	0.850
Female sex (vs. male)	0.75 (0.51–1.10)	0.137	0.70 (0.45–1.10)	0.122
Rural residence (vs. urban)	1.04 (0.60–1.79)	0.894	0.88 (0.48–1.61)	0.671
Smoking (yes vs. no)	1.10 (0.61–1.98)	0.751	0.73 (0.37–1.43)	0.357
Exacerbation frequency ≥2/year	2.20 (1.41–3.44)	<0.001	2.41 (1.47–3.95)	<0.001
Exacerbation frequency 1 every 2–3 years or less frequently	0.29 (0.13–0.65)	0.003	0.24 (0.09–0.61)	0.003
Previous IBD-related surgery	0.92 (0.59–1.44)	0.716	0.92 (0.52–1.62)	0.769
5-ASA therapy	1.74 (1.05–2.91)	0.033	1.78 (0.96–3.32)	0.067
Corticosteroid therapy	1.51 (0.95–2.38)	0.079	1.77 (1.03–3.05)	0.039
Immunosuppressive therapy	0.80 (0.47–1.38)	0.431	0.46 (0.24–0.89)	0.021
Biologic therapy	1.14 (0.74–1.76)	0.549	0.99 (0.57–1.70)	0.966
L2 colonic disease vs. L1 ileal disease	1.77 (1.03–3.05)	0.040	2.32 (1.25–4.30)	0.008
L3 ileocolonic disease vs. L1 ileal disease	1.87 (1.13–3.11)	0.016	2.24 (1.25–4.04)	0.007
L4 upper GI involvement vs. L1 ileal disease	0.44 (0.10–1.95)	0.279	0.47 (0.08–2.83)	0.412
Any intestinal complication	2.09 (0.89–4.86)	0.089	2.49 (0.87–7.10)	0.088
Extraintestinal manifestations	2.88 (1.76–4.71)	<0.001	2.88 (1.68–4.93)	<0.001

## Data Availability

The datasets analyzed during the current study are not publicly available due to institutional and patient privacy restrictions but are available from the corresponding author on reasonable request.

## Supplementary Materials

The following supporting information can be downloaded at: https://www.mdpi.com/article/10.3390/jcm15176851/s1, Table S1: Sensitivity analysis of the multivariable logistic regression models for active disease using Firth penalized logistic regression (Panel A and B); Table S2: Sensitivity analysis of factors associated with active Crohn’s disease after exclusion of extraintestinal manifestations from the multivariable model; Table S3: Sensitivity analysis of multivariable logistic regression models after exclusion of smoking status as a covariate (Panel A and B).

## Abbreviations

The following abbreviations are used in this manuscript:

5-ASA	5-Aminosalicylic acid
aOR	Adjusted odds ratio
AUC	Area under the receiver operating characteristic curve
BMI	Body mass index
CD	Crohn’s disease
CI	Confidence interval
CRP	C-reactive protein
df	Degrees of freedom
ENEIDA	Nationwide Study on Genetic and Environmental Determinants of Inflammatory Bowel Disease
GI	Gastrointestinal
GIVES-21	Global IBD Visualization of Epidemiology Studies in the 21st Century
HBI	Harvey–Bradshaw Index
IBD	Inflammatory bowel disease
ICH-GCP	International Council for Harmonisation–Good Clinical Practice
IQR	Interquartile range
LEC	Local Ethics Committee
OR	Odds ratio
SWIBREG	Swedish Inflammatory Bowel Disease Register
