# Supplementary material for: Inflammatory Bowel Disease in Kazakhstan: Phenotypes, Disease Activity, and Treatment Patterns from a National Registry
Source: J Clin Med. 2026 Sep 4;15(17):6851. doi: 10.3390/jcm15176851 (PMC13566639; doi:10.3390/jcm15176851)
Supplement: Supplementary file 1 [file jcm-15-06851-s001.zip › jcm-4492834-supplementary.pdf]

**Supplementary Table S1. Sensitivity analysis of the multivariable logistic regression models for active disease using Firth penalized logistic regression.**

**Panel A. Ulcerative colitis**

| Predictor | Conventional aOR (95% CI)* | p-value | Firth OR (95% CI)** | p-value |
|---|---|---|---|---|
| Age | 1.00 (0.99–1.01) | 0.841 | 1.00 (0.99–1.01) | 0.843 |
| BMI | 0.97 (0.93–1.01) | 0.190 | 0.97 (0.93–1.01) | 0.190 |
| Disease duration | 0.99 (0.97–1.01) | 0.474 | 0.99 (0.97–1.01) | 0.451 |
| Female sex | 0.87 (0.61–1.25) | 0.450 | 0.87 (0.61–1.24) | 0.452 |
| Rural residence | 2.11 (1.25–3.56) | 0.005 | 2.05 (1.25–3.50) | 0.004 |
| Smoking | 1.33 (0.68–2.60) | 0.404 | 1.29 (0.69–2.58) | 0.434 |
| Exacerbations ≥2/year | 3.47 (2.18–5.51) | <0.001 | 3.35 (2.15–5.39) | <0.001 |
| 1 every 2–3 years or less frequently | 0.41 (0.23–0.73) | 0.002 | 0.42 (0.24–0.74) | 0.003 |
| Previous IBD-related surgery | 0.74 (0.31–1.77) | 0.492 | 0.73 (0.32–1.77) | 0.480 |
| 5-ASA therapy | 1.21 (0.45–3.27) | 0.706 | 1.26 (0.45–3.14) | 0.641 |
| Corticosteroid therapy | 5.96 (3.04–11.69) | <0.001 | 5.56 (3.02–11.35) | <0.001 |
| Immunosuppressive therapy | 2.71 (1.15–6.38) | 0.022 | 2.53 (1.17–6.24) | 0.017 |
| Biologic therapy | 0.73 (0.46–1.16) | 0.188 | 0.74 (0.47–1.17) | 0.190 |
| **E1 vs E2** | 1.48 (0.91–2.41) | 0.111 | 1.46 (0.91–2.38) | 0.115 |
| **E3 vs E2** | 1.01 (0.69–1.48) | 0.972 | 1.01 (0.69–1.47) | 0.978 |
| Any intestinal complication | 2.70 (0.70–10.46) | 0.151 | 2.32 (0.73–9.77) | 0.164 |
| Extraintestinal manifestations | 1.31 (0.79–2.15) | 0.298 | 1.29 (0.80–2.14) | 0.309 |

**Panel B. Crohn's disease**

| Predictor | Conventional aOR (95% CI) | p-value | Firth OR (95% CI) | p-value |
|---|---|---|---|---|
| Age | 0.99 (0.97–1.00) | 0.136 | 0.99 (0.98–1.00) | 0.141 |
| BMI | 0.97 (0.92–1.01) | 0.172 | 0.97 (0.93–1.01) | 0.179 |
| Disease duration | 1.00 (0.98–1.02) | 0.850 | 1.00 (0.98–1.02) | 0.893 |
| Female sex | 0.70 (0.45–1.10) | 0.122 | 0.71 (0.46–1.10) | 0.128 |
| Rural residence | 0.88 (0.48–1.61) | 0.671 | 0.88 (0.49–1.61) | 0.669 |
| Smoking | 0.73 (0.37–1.43) | 0.357 | 0.73 (0.38–1.43) | 0.356 |
| Exacerbations ≥2/year | 2.41 (1.47–3.95) | <0.001 | 2.31 (1.44–3.77) | <0.001 |
| 1 every 2–3 years or less frequently | 0.24 (0.09–0.61) | 0.003 | 0.26 (0.10–0.63) | 0.003 |
| Previous IBD-related surgery | 0.92 (0.52–1.62) | 0.769 | 0.92 (0.53–1.61) | 0.758 |
| 5-ASA therapy | 1.78 (0.96–3.32) | 0.067 | 1.74 (0.95–3.17) | 0.074 |
| Corticosteroid therapy | 1.77 (1.03–3.05) | 0.039 | 1.71 (1.02–2.95) | 0.042 |
| Immunosuppressive therapy | 0.46 (0.24–0.89) | 0.021 | 0.47 (0.25–0.91) | 0.024 |
| Biologic therapy | 0.99 (0.57–1.70) | 0.966 | 0.98 (0.58–1.68) | 0.952 |
| L2 vs L1 | 2.32 (1.25–4.30) | 0.008 | 2.24 (1.23–4.11) | 0.009 |
| L3 vs L1 | 2.24 (1.25–4.04) | 0.007 | 2.17 (1.22–3.86) | 0.008 |
| L4 vs L1 | 0.47 (0.08–2.83) | 0.412 | 0.52 (0.09–2.71) | 0.438 |
| Any intestinal complication | 2.49 (0.87–7.10) | 0.088 | 2.26 (0.87–6.58) | 0.095 |
| Extraintestinal manifestations | 2.88 (1.68–4.93) | <0.001 | 2.72 (1.64–4.67) | <0.001 |

*Conventional logistic regression estimates are presented with Wald 95% confidence intervals and Wald p-values.

**Firth estimates, confidence intervals, and p-values were obtained using penalized-likelihood procedures. For each disease, the conventional and Firth models were fitted to the same complete-case analytical sample and included the same prespecified covariates.
Panel A included 981 patients with ulcerative colitis (UC), and Panel B included 472 patients with Crohn's disease (CD). In Panel A, the prespecified covariates were age, sex, body mass index (BMI), residence, smoking status, disease duration, exacerbation frequency, previous IBD-related surgery, current 5-aminosalicylic acid (5-ASA), corticosteroid, immunosuppressive and biologic therapy, UC extent according to the Montreal classification (E1–E3), intestinal complications, and extraintestinal manifestations (EIMs). In Panel B, the same covariates were included, with CD location according to the Montreal classification (L1–L4) instead of UC extent. The conventional and Firth penalized logistic regression models were fitted to the same complete-case analytical sample within each disease group.
**Abbreviations:** aOR, adjusted odds ratio; OR, odds ratio; CI, confidence interval; BMI, body mass index; IBD, inflammatory bowel disease; 5-ASA, 5-aminosalicylic acid; EIM, extraintestinal manifestation; UC, ulcerative colitis; CD, Crohn's disease; E1, proctitis; E2, left-sided colitis; E3, extensive colitis; L1, ileal disease; L2, colonic disease; L3, ileocolonic disease; L4, upper gastrointestinal involvement.

**Supplementary Table S2. Sensitivity analysis of factors associated with active Crohn's disease after exclusion of extraintestinal manifestations from the multivariable model.**

| Predictor | Primary model aOR (95% CI) | p-value | Model excluding EIM aOR (95% CI) | p-value |
|---|---|---|---|---|
| Age, per 1-year increase | 0.99 (0.97–1.00) | 0.136 | 0.99 (0.97–1.00) | 0.103 |
| BMI, per 1 kg/m² increase | 0.97 (0.92–1.01) | 0.172 | 0.97 (0.92–1.01) | 0.132 |
| Disease duration, per 1-year increase | 1.00 (0.98–1.02) | 0.850 | 1.00 (0.98–1.02) | 0.785 |
| Female sex (vs male) | 0.70 (0.45–1.10) | 0.122 | 0.70 (0.45–1.09) | 0.115 |
| Rural residence (vs urban) | 0.88 (0.48–1.61) | 0.671 | 0.90 (0.49–1.63) | 0.725 |
| Smoking (yes vs no) | 0.73 (0.37–1.43) | 0.357 | 0.79 (0.41–1.51) | 0.476 |
| Exacerbation frequency ≥2/year (vs 0–1/year) | 2.41 (1.47–3.95) | <0.001 | 2.42 (1.49–3.93) | <0.001 |
| Exacerbation frequency 1 every 2–3 years or less frequently (vs 0–1/year) | 0.24 (0.09–0.61) | 0.003 | 0.26 (0.11–0.64) | 0.003 |
| Previous IBD-related surgery (yes vs no) | 0.92 (0.52–1.62) | 0.769 | 0.87 (0.49–1.52) | 0.613 |
| 5-ASA therapy (yes vs no) | 1.78 (0.96–3.32) | 0.067 | 1.77 (0.97–3.25) | 0.063 |
| Corticosteroid therapy (yes vs no) | 1.77 (1.03–3.05) | 0.039 | 1.95 (1.15–3.32) | 0.013 |
| Immunosuppressive therapy (yes vs no) | 0.46 (0.24–0.89) | 0.021 | 0.55 (0.29–1.05) | 0.069 |
| Biologic therapy (yes vs no) | 0.99 (0.57–1.70) | 0.966 | 1.05 (0.62–1.79) | 0.854 |
| L2 colonic disease (vs L1 ileal disease) | 2.32 (1.25–4.30) | 0.008 | 2.30 (1.25–4.21) | 0.007 |
| L3 ileocolonic disease (vs L1 ileal disease) | 2.24 (1.25–4.04) | 0.007 | 2.31 (1.30–4.12) | 0.004 |
| L4 upper gastrointestinal involvement (vs L1 ileal disease) | 0.47 (0.08–2.83) | 0.412 | 0.43 (0.08–2.42) | 0.338 |
| Any intestinal complication (yes vs no) | 2.49 (0.87–7.10) | 0.088 | 2.72 (0.97–7.62) | 0.056 |
| Extraintestinal manifestations (yes vs no) | 2.88 (1.68–4.93) | <0.001 | — | — |

The analysis included 472 patients with Crohn's disease, corresponding to the same complete-case analytical sample used in the primary multivariable model. The primary model included age, sex, body mass index (BMI), residence, smoking status, disease duration, exacerbation frequency, previous IBD-related surgery, current 5-aminosalicylic acid (5-ASA), corticosteroid, immunosuppressive and biologic therapy, Crohn's disease location according to the Montreal classification (L1-L4), intestinal complications,

and extraintestinal manifestations (EIMs). The sensitivity model retained the same analytical sample and all covariates except EIMs.

**Abbreviations:** aOR, adjusted odds ratio; CI, confidence interval; BMI, body mass index; IBD, inflammatory bowel disease; 5-ASA, 5-aminosalicylic acid; EIM, extraintestinal manifestation; L1, ileal disease; L2, colonic disease; L3, ileocolonic disease; L4, upper gastrointestinal involvement.

**Supplementary Table S3. Sensitivity analysis of multivariable logistic regression models after exclusion of smoking status as a covariate**

**Panel A. Ulcerative colitis**

| Predictor | Primary model, aOR (95% CI) | p-value | Model excluding smoking status, aOR (95% CI) | p-value |
|---|---|---|---|---|
| Age | 1.00 (0.99–1.01) | 0.841 | 1.00 (0.99–1.01) | 0.992 |
| BMI | 0.97 (0.93–1.01) | 0.190 | 0.98 (0.94–1.02) | 0.349 |
| Disease duration | 0.99 (0.97–1.01) | 0.474 | 0.99 (0.97–1.01) | 0.462 |
| Female sex | 0.87 (0.61–1.25) | 0.450 | 0.84 (0.59–1.18) | 0.307 |
| Rural residence | 2.11 (1.25–3.56) | 0.005 | 2.24 (1.36–3.71) | 0.002 |
| Smoking | 1.33 (0.68–2.60) | 0.404 | — | — |
| Exacerbations ≥2/year | 3.47 (2.18–5.51) | <0.001 | 3.41 (2.15–5.41) | <0.001 |
| 1 every 2–3 years or less frequently | 0.41 (0.23–0.73) | 0.002 | 0.51 (0.30–0.89) | 0.017 |
| Previous IBD-related surgery | 0.74 (0.31–1.77) | 0.492 | 0.71 (0.30–1.67) | 0.433 |
| 5-ASA therapy | 1.21 (0.45–3.27) | 0.706 | 1.08 (0.41–2.85) | 0.871 |
| Corticosteroid therapy | 5.96 (3.04–11.69) | <0.001 | 6.30 (3.23–12.29) | <0.001 |
| Immunosuppressive therapy | 2.71 (1.15–6.38) | 0.022 | 2.56 (1.10–5.97) | 0.030 |
| Biologic therapy | 0.73 (0.46–1.16) | 0.188 | 0.76 (0.49–1.20) | 0.244 |
| E1 vs E2 | 1.48 (0.91–2.41) | 0.111 | 1.51 (0.94–2.42) | 0.089 |
| E3 vs E2 | 1.01 (0.69–1.48) | 0.972 | 1.00 (0.69–1.46) | 1.000 |
| Any intestinal complication | 2.70 (0.70–10.46) | 0.151 | 2.43 (0.64–9.28) | 0.193 |
| Extraintestinal manifestations | 1.31 (0.79–2.15) | 0.298 | 1.24 (0.76–2.04) | 0.393 |

**Panel B. Crohn's disease**

| Predictor | Primary model, aOR (95% CI) | p-value | Model excluding smoking status, aOR (95% CI) | p-value |
|---|---|---|---|---|
| Age | 0.99 (0.97–1.00) | 0.136 | 0.99 (0.97–1.00) | 0.082 |
| BMI | 0.97 (0.92–1.01) | 0.172 | 0.98 (0.93–1.02) | 0.285 |
| Disease duration | 1.00 (0.98–1.02) | 0.850 | 1.00 (0.98–1.02) | 0.833 |
| Female sex | 0.70 (0.45–1.10) | 0.122 | 0.75 (0.48–1.16) | 0.195 |
| Rural residence | 0.88 (0.48–1.61) | 0.671 | 0.90 (0.49–1.64) | 0.731 |
| Smoking | 0.73 (0.37–1.43) | 0.357 | — | — |
| Exacerbations ≥2/year | 2.41 (1.47–3.95) | <0.001 | 2.36 (1.45–3.85) | <0.001 |
| 1 every 2–3 years or less frequently | 0.24 (0.09–0.61) | 0.003 | 0.23 (0.09–0.57) | 0.002 |
| Previous IBD-related surgery | 0.92 (0.52–1.62) | 0.769 | 0.89 (0.50–1.57) | 0.682 |
| 5-ASA therapy | 1.78 (0.96–3.32) | 0.067 | 1.82 (0.98–3.37) | 0.056 |
| Corticosteroid therapy | 1.77 (1.03–3.05) | 0.039 | 1.77 (1.03–3.04) | 0.038 |
| Immunosuppressive therapy | 0.46 (0.24–0.89) | 0.021 | 0.47 (0.25–0.92) | 0.026 |

| | | | | |
|---|---|---|---|---|
| Biologic therapy | 0.99 (0.57–1.70) | 0.966 | 1.06 (0.62–1.81) | 0.837 |
| L2 vs L1 | 2.32 (1.25–4.30) | 0.008 | 2.18 (1.19–3.99) | 0.011 |
| L3 vs L1 | 2.24 (1.25–4.04) | 0.007 | 2.16 (1.22–3.83) | 0.009 |
| L4 vs L1 | 0.47 (0.08–2.83) | 0.412 | 0.48 (0.08–2.77) | 0.409 |
| Any intestinal complication | 2.49 (0.87–7.10) | 0.088 | 2.16 (0.81–5.79) | 0.126 |
| Extraintestinal manifestations | 2.88 (1.68–4.93) | <0.001 | 2.82 (1.65–4.81) | <0.001 |

The primary complete-case models included 981 patients with ulcerative colitis (UC) and 472 patients with Crohn's disease (CD). After excluding smoking status as a covariate, the sensitivity models included the full disease-specific cohorts (UC, n=1,041; CD, n=480). All other prespecified covariates were retained.

**Abbreviations:** aOR, adjusted odds ratio; CI, confidence interval; BMI, body mass index; IBD, inflammatory bowel disease; 5-ASA, 5-aminosalicylic acid; EIM, extraintestinal manifestation; UC, ulcerative colitis; CD, Crohn's disease; E1, proctitis; E2, left-sided colitis; E3, extensive colitis; L1, ileal disease; L2, colonic disease; L3, ileocolonic disease; L4, upper gastrointestinal involvement.
